# Investigation into Ultrasonic Oscillation-Assisted Nickel Electroplating onto a Diamond Surface

**DOI:** 10.3390/mi16080962

**Published:** 2025-08-21

**Authors:** Qingming Fan, Bin Guo, Guokang Su, Hui Qi, Pengfan Li, Chuanyun Zhang, Kai Cheng

**Affiliations:** 1 School of Mechatronic Engineering, Xi’an Technological University, Xi’an 710021, China; fanqingming@xatu.edu.cn (Q.F.); suguokang@xatu.edu.cn (G.S.); lpf9697@163.com (P.L.); 2 Shaanxi Engineering Research Center of Digital Precision Electrochemical Machining, Xi’an 710021, China; 3 School of Electro-Mechanical Engineering, Guangdong University of Technology, Guangzhou 510006, China; 19917991796@163.com; 4 College of Chemistry and Materials, Taiyuan Normal University, Jinzhong 030619, China; qihui@xatu.edu.cn; 5 College of Engineering, Design and Physical Sciences, Brunel University London, London UB8 3PH, UK

**Keywords:** ultrasonic assisted electroplating, nickel electroplating, diamond surface, weight-gain rate

## Abstract

At present, there are some challenging issues for diamond electroplating devices, such as poor particle–cathode contact uniformity, low conductivity, inefficient deposition, and complex disassembly/cleaning process of the device. To overcome these issues, an ultrasonic oscillation-assisted nickel electroplating device is innovatively designed and presented in this paper. The device features: (1) innovative architecture enabling rapid disassembly; (2) ultrasonic enhancement of diamond particle mobility (frequency × amplitude); (3) optimized electrical contact interfaces. In this paper, the effects of electroplating current, output power of ultrasonic oscillator and diamond particle size on nickel electroplating onto diamond surface are further studied particularly by ultrasonic assisted electroplating. The experimental results show that the ultrasonic oscillation assisted electroplating greatly improves the uniformity of the coating on the diamond surface and effectively prevents the adhesion between diamond particles. While the process parameters are electroplating current of 3 A, output power of ultrasonic oscillator 900 W, diamond particle size of 120/140, the weight-gain rate is 20.6%, the nickel content of the coating reaches 81.95%, and the coating is excellent uniformed without agglomeration. The research presented provides fundamental understanding for further development and application of ultrasonic oscillation-assisted electroplating technology particularly for broad precision engineering purposes.

## 1. Introduction

Many studies have demonstrated that coating a metallic layer onto the surface of diamond particles can significantly improve the bonding strength between the diamond particles and the substrate in diamond tools [1,2]. This coating also provides effective oxygen isolation protection and reduces thermal damage to the diamond, and consequently extends the service life of diamond tools [3,4]. Common surface-coating techniques for diamonds include electroplating [5], salt bath plating [6], chemical plating [7], physical vapor deposition (PVD) [8], and chemical vapor deposition (CVD) [9]. Diamond surface electroplating utilizes electrochemical principles to reduce metal ions in the plating solution, depositing metallic elements onto the diamond surface to form a uniform coating. However, due to the non-conductive nature of diamond particles’ surfaces, direct electroplating is not feasible. To overcome this limitation, an initial conductive layer must be applied using electroless plating or alternative techniques. Once the surface becomes conductive, electroplating can be carried out to further thicken the coating or apply additional metallic layers [5]. Owing to its high-temperature resistance, oxidation resistance, excellent mechanical strength, ductility, and corrosion resistance, nickel is widely utilized as a coating material for diamond surfaces. In the application of metal plating on the surface of diamond abrasive particles, nickel plating is the most widely used.

The application of ultrasonic technology in traditional electroplating can enhance the ion deposition process, refine grain size, and improve the quality of the coating [10,11]. Vasudevan applied ultrasonic waves with a frequency of 22 kHz and a power range of 0–500 W during the Watts bath nickel electroplating process. The experimental results showed that the use of ultrasonic waves could reduce hydrogen evolution and improve deposition rate [12]. P. B. S. N. V. Prasad discovered that the ultrasonic method increased the deposition rate of nickel electroplating, while also making the coating surface smoother and improving microhardness [13]. Jensen investigated the mechanism of high-frequency ultrasonic nickel electroplating, revealing that in Watts bath the application of 1 MHz ultrasonic waves achieved uniform nickel coating thickness on both the bottom and side walls of three-dimensional grooves [14]. Yu introduced an ultrasonic element during a pulse plating process to prepare NiFeP alloy films, and obtained the NiFeP films with smaller grain size and smooth surface [15]. Yu obtained the multilayered Ni coatings by introducing the periodic ultrasonic wave into the ordinary electroplating process [16]. Ban et al. used ultrasonic irradiation (1 kHz) with a different frequency to electroplated Ni coating on a carbon steel substrate to protect carbon steel. The results showed that, compared to that without ultrasonic irradiation imposition, ultrasonic irradiation during electroplating could refine the coating grain and decrease the micro-pores in the coating, resulting in improvement of the coating corrosion and hardness [17]. Zhao investigated the effect of ultrasonic activation on the bonding strength of electroplating nanocrystalline Ni coatings. Under ultrasonic conditions (200 W, 80 kHz), the bonding strength was increased by 60.7% compared to conventional plating without ultrasonic oscillation [18]. The above research has fully demonstrated that ultrasonic oscillation-assisted nickel electroplating is extremely beneficial.

At present, research on electroplating technology for diamond surfaces primarily focuses on improving plating methods and equipment. For example, Selvam developed a 200 mL miniature closed drum electroplating apparatus that enables uniform and dense deposition of a nickel–tungsten coating on the diamond surface, effectively filling micro-cracks and thereby enhancing the utilization efficiency of diamond particles [19]. Zhang proposed a method of barrel plating on a diamond surface on a rotating electrode, developed the corresponding barrel plating device, and studied the effects of plating parameters on the deposition rate and morphology of diamond coatings [20]. Feng optimized the diamond-loading position in traditional barrel plating devices by incorporating a cathode-rotation function, which increased the rolling frequency of the abrasive pile during electroplating. This modification improved the overall contact uniformity between abrasive particles and the electrode, enhanced the electrical conductivity of the abrasive particles, and consequently improved the stability of the plating process and the quality of the resulting coating [21]. Sun designed a channel-shaped metal disc electrode that significantly improved the electrical connection between diamond particles and the cathode, thereby enhancing conductivity. Using this method, nickel plating was successfully applied to diamond surfaces, achieving nanoparticles with sizes ranging from 200 to 250 μm and producing a uniform and dense nickel coating [22]. In addition, researchers have investigated key factors influencing nickel plating on diamond surfaces and used machine learning techniques to predict quality. Fang discussed the effect of electroplating current on both the nickel plating rate and toughness of the coating [23]. Deng studied the influence of the additive 1,4-bis-2-butyne on the properties of nickel-based composite coatings using a sulfonate nickel plating solution [24]. Huang prepared Ni/diamond coatings using a nickel sulfate electroplating solution and explored how variations in plating solution composition and electroplating parameters affect the quality of the nickel coating on diamond surfaces [25]. Sun employed machine learning techniques to develop models capable of predicting defects, coating color, and coating mass for an electroplating process to achieve high-quality electroplated coatings [26]. Wu developed several Machine Learning (ML) models; these models were inspected with explainable artificial intelligence (XAI) methods [27].

Although numerous scientific researchers have conducted extensive studies on diamond electroplating, several challenges remain in the current process, including poor uniformity of contact between diamond particles and the cathode, low electrical conductivity, and limited plating efficiency. Additionally, the procedures involved in the assembly, disassembly, and cleaning of electroplating equipment remain complex and cumbersome. To solve the above problems, this study innovatively proposes the first design of a device using ultrasonic oscillation-assisted nickel electroplating onto the diamond surface. The device not only simplifies the processes of assembly, disassembly, and cleaning but also employs ultrasonic oscillation to enhance the movement frequency and amplitude of diamond particles during electroplating. This ultimately improves the electrical conductivity between diamond particles and the uniformity of their contact with the cathode, leading to a significant enhancement in coating quality. This work provides a theoretical foundation for enhancing the surface quality of diamond in ultrasonically assisted electroplating.

## 2. Nickel Electroplating Experiment

### 2.1. Mechanism of Ultrasonic Oscillation-Assisted Nickel Electroplating onto the Diamond Surface

Ultrasonic oscillation-assisted electroplating applies the cavitation effect and oscillation effect of ultrasonic oscillators to electroplating. In ultrasonic oscillators, water is affected by strong changes in sound pressure, resulting in the generation of tiny bubble cavitation effects. Small bubbles rapidly increase and burst under the action of sound pressure, causing strong vibrations in water. Vibration propagates through sound radiation, producing an oscillation effect. Under the combined effects of cavitation and oscillation, the following functions have been produced on electroplating. Firstly, it enhances the diffusion rate of the electroplating solution, thereby increasing the plating rate, reducing or eliminating concentration polarization, and facilitating the deposition of metals that are otherwise difficult to plate under normal conditions. Secondly, it minimizes material agglomeration during the electroplating process, resulting in a more uniform distribution of plating materials within the solution and thus improving the overall quality of the coating. Additionally, the cavitation effect generated by the ultrasonic waves produces micro-jets that continuously clean the electrode surface. This action reduces or prevents electrode passivation, thereby accelerating both the anodic oxidation and cathodic reduction reactions, which in turn enhances the deposition efficiency. Furthermore, the ultrasonic oscillator helps remove hydrogen adsorbed on the electrode surface, thereby mitigating issues such as hydrogen embrittlement and surface mottling, and decreasing internal stress within the coating [28,29,30].

The oscillation function of the ultrasonic oscillator facilitates the movement of diamond particles within the electroplating solution, resulting in a more uniform distribution of these particles. This improvement enhances the uniformity of contact between the diamond particles and the electroplating cathode, increases the interaction opportunities between the diamond surfaces and metal ions in the solution, and ultimately improves both the uniformity and overall quality of the deposited coating. During the electroplating process, diamond particles tend to accumulate on the cathode due to gravitational forces. Through inter-particle contact and conduction, a conductive path is formed between the particles and the cathode, thereby establishing a closed circuit within the plating system. Under the influence of the ultrasonic oscillator, the continuous movement of diamond particles is maintained, enabling a more uniform and efficient nickel electroplating process.

(1) Anode Reaction

During the nickel electroplating on the surface of diamond, the nickel metal serves as the anode and oxidizes to Ni^2+^ ions (Equation (1)), which dissolve into the electrolyte. At same time, side reactions commonly occur at the anode; oxygen evolution takes place at the anode (Equation (2)). Concurrently, the anode nickel metal undergoes oxidation to form Ni_2_O_3_ (Equation (3)). This forms a passivating oxide film that inhibits further nickel dissolution.

(1) $$\mathrm{Ni}\;-\;2e^{-}\to Ni^{2+}$$

(2) $$4OH^{-}\;-\;2e\;\to 2H_{2}O+O_{2}↑$$

(3) $$4\mathrm{Ni}\;+\;{3O}_{2}\to 2Ni_{2}O_{3}$$

(2) Cathode reaction

Under the influence of the electric field, Ni^2+^ ions in the electroplating solution mi-grate toward the cathode, where they undergo reduction to metallic nickel. This deposited nickel forms a uniform coating on the previous electroless nickel-plated diamonds (Equation (4)). Meanwhile, a side reaction occurs at the cathode: water electrolysis generates hydrogen gas via proton reduction (Equation (5)).

(4) $$Ni^{2+}+2e\;\to \mathrm{Ni}$$

(5) $$2H^{+}+2e\;\to H_{2}↑$$

### 2.2. Device of Ultrasonic Oscillation-Assisted Nickel Electroplating on Diamond Surface

**Figure 1:**
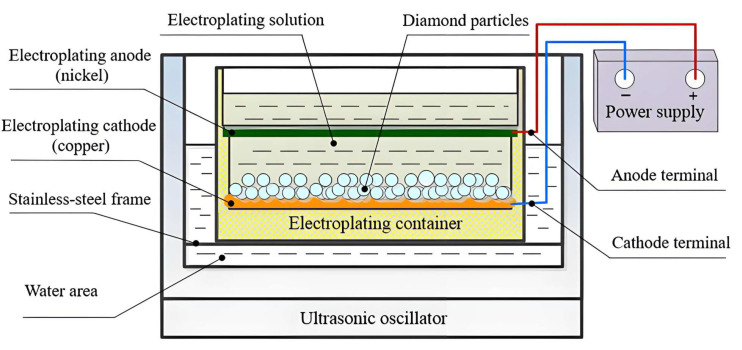
Schematic diagram of ultrasonic assisted nickel electroplating on diamond surface.

The designed device of ultrasonic oscillation-assisted nickel electroplating is mainly composed of an ultrasonic oscillator, an electroplating power supply, and an electroplating container, as shown in Figure 1. The ultrasonic oscillator operates at a frequency of 40 kHz with an output power of 900 W. It features adjustable settings and incorporates a heating function, capable of reaching a maximum temperature of 100 °C. The electroplating power supply is a DC pulse power supply with continuously adjustable output voltage ranging from 0 to 30 V and an output current ranging from 0 to 20 A. This power supply supports two operational modes—constant voltage and constant current—thereby fulfilling the requirements of the electroplating process.

**Figure 2:**
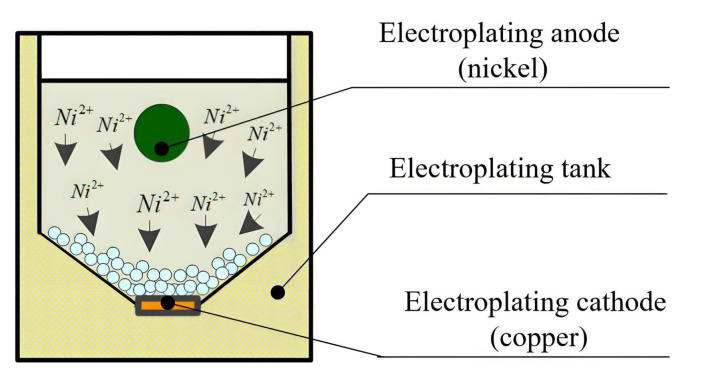
The electroplating container.

The electroplating container includes components such as the electroplating tank, an electroplating anode, and an electroplating cathode, as shown in Figure 2. The design principles of the electroplating tank aim to ensure sufficient structural strength to allow the penetration of ultrasonic waves, ease of installation and removal of the electrodes, excellent electrical conductivity, and resistance to high temperatures. To enable visual monitoring of the electroplating solution and diamond particles during the process, the electroplating tank is constructed from acrylic sheets, which provide transparency, corrosion resistance, and insulation properties. Grooves are also provided on both sides inside the electroplating tank for fixing the nickel rod used as the electroplating anode. A notch is set at the bottom of the electroplating tank to fix the copper plate as the electroplating cathode. The bottom of the electroplating tank is designed as an inclined plane to increase the contact area between the diamond particles and the cathode by increasing the area so as to improve the nickel electroplating rate on the diamond surface. Two threaded holes are drilled at the position corresponding to the anode and cathode on the outside of the electroplating tank for connecting the anode terminal and the cathode terminal.

### 2.3. Experiment Scheme

The electroplating current and the diamond particles size directly influence the quality and thickness of the nickel coating. Adjusting the output power of the ultrasonic oscillator can change the intensity of the force acting on the diamond surface, thereby affecting the movement and distribution of diamond particles in the electroplating solution, influencing the interaction between nickel ions and diamond particles during the electroplating process, and ultimately affecting the quality and thickness of the final coating. To systematically evaluate these effects, a single-factor experimental design is employed to investigate the influence of electroplating current, ultrasonic oscillator output power and diamond particle size on the nickel coating of the diamond surfaces by the ultrasonic oscillation-assisted nickel electroplating. The process parameters are shown in Table 1.

The experiment process is as follows:

(1) Inject water into the ultrasonic oscillator, and then place the electroplating container on the stainless-steel frame inside the ultrasonic oscillator.
(2) Pour the prepared electroplating solution (the components are shown in Table 2) into the electroplating tank, with the liquid level ensuring immersion of the electroplating anode.
(3) Place a certain quantity of diamond particles that have undergone electroless nickel plating into the electroplating solution. Under the action of gravity, diamond particles accumulate and completely cover the electroplating cathode. Turn on the ultrasonic oscillator and oscillate for 10 min to evenly disperse and fully wet the diamond particles in the electroplating solution.
(4) The anode and the cathode of the electroplating tank are, respectively, connected to the positive pole and negative pole of the electroplating power supply.
(5) Heating and oscillating the water area of the ultrasonic oscillator provides a suitable temperature for the electroplating solution, and causes the diamond particles in the electroplating solution to move, thus forming an ultrasonic oscillation electroplating system.
(6) Adjust the electroplating current, the output power of the ultrasonic oscillator, and the diamond particle size to the electroplate nickel on the diamond surface. After electroplating for a certain duration, take out the diamond treated with nickel electroplating and clean, dry, weigh, and test the coating.

**Table 2:** The components of electroplating solutions.

Component	Medicine	Concentration
Metal salt	Nickel sulfate (NiSO_4_)	250 g/L
Activator of the anode	Nickel Chloride (NiCl_2_)	40 g/L
Buffer agent	Boric acid	20 g/L
Wetting agent	Sodium dodecyl sulfate (SDS)	0.05 g/L

## 3. Results and Discussion

### 3.1. The Influence of Electroplating Current on Nickel Electroplating onto Diamond Surface

Single-factor experiments are conducted to investigate the influence of the electroplating current on the weight-gain rate of the coating and agglomeration phenomena of diamond surfaces. The experimental conditions are as follows: diamond-loading capacity of 2 g, electroplating temperature of 50 °C, electroplating currents of 1 A, 3 A, 5 A, and 7 A, ultrasonic oscillator output power of 900 W, diamond particle size of 70/80, and electroplating duration of 3 h.

**Figure 3:**
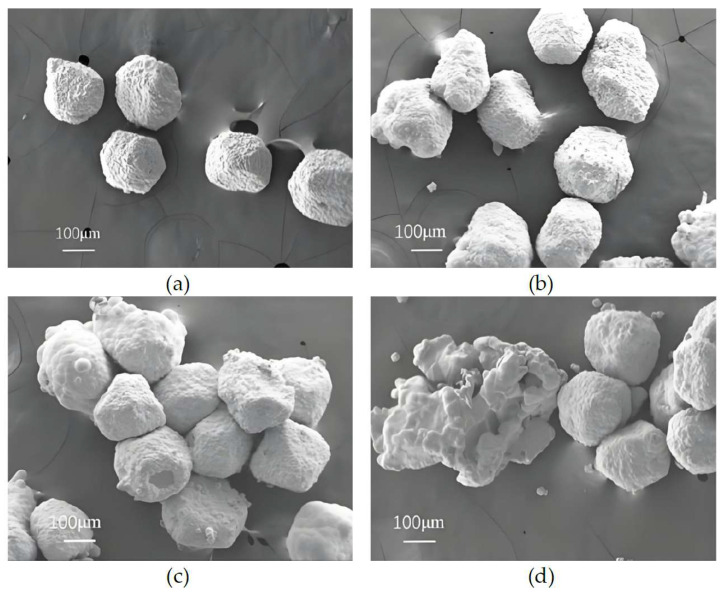
SEM images of nickel electroplating diamond under varying electroplating current conditions: (**a**) 1 A; (**b**) 3 A; (**c**) 5 A; (**d**) 7 A.

Figure 3 presents SEM images of nickel-plated diamond particles under varying electroplating current conditions. It can be observed from Figure 3 that the diamond surface is uniformly coated with a layer of metal particles across different current levels. The primary distinction lies in the fact that as the electroplating current increases, diamond particles tend to exhibit caking and adhesion phenomena. Specifically, Figure 3a,b demonstrate that when the electroplating current is set at 1 A and 3 A, respectively, the nickel coating is evenly distributed without any signs of caking and adhesion phenomena. Furthermore, with an increase in current density, the coating thickness gradually increases. This phenomenon can be attributed to the relatively slow reduction rate of nickel ions in the electroplating solution under lower current conditions, resulting in a slower deposition rate of metallic nickel. Additionally, due to the action of the ultrasonic oscillator, the diamond particles remained in continuous motion throughout the electroplating process, thereby preventing particle agglomeration. However, when the current reaches 5 A, as shown in Figure 3c, the coating thickness of the diamond particles significantly increases. When the current further increases to 7 A, as illustrated in Figure 3d, significant agglomeration and lump formation of the diamond particles become evident. This is primarily due to the increased deposition rate on the diamond surface per unit time caused by the higher electroplating current, while the movement amplitude of the diamond particles remains relatively limited, ultimately leading to the adhesion and clumping phenomena of diamond particles.

**Figure 4:**
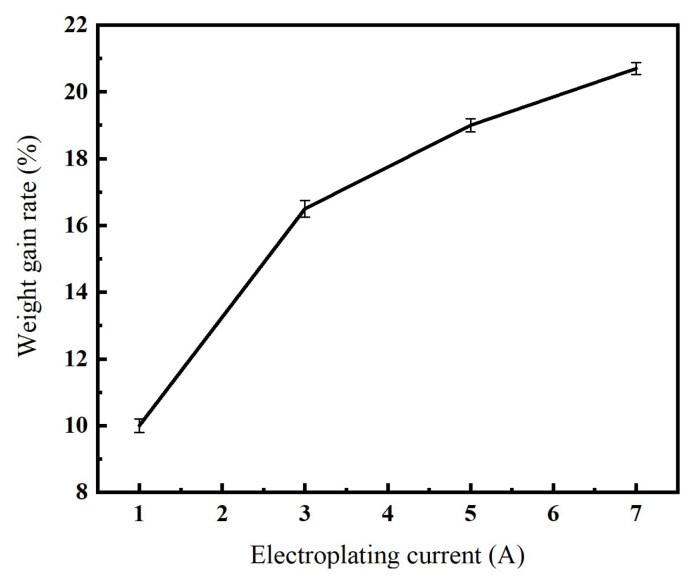
Relationship between electroplating current and coating weight-gain rate.

Figure 4 illustrates the effect of electroplating current on the weight-gain rate of the nickel-coated diamond. As shown in the figure, the weight-gain rate increases with the electroplating current. This occurs primarily because a higher electroplating current releases more electrons per unit time at the cathode, thereby accelerating nickel deposition around the diamond particles and consequently increasing the overall weight-gain rate. Additionally, it can be observed that the growth rate of the weight-gain rate initially increases and subsequently decreases with further increases in electroplating current. The initial increase occurs because more metal ions participate effectively in the electrochemical reaction under stronger electric field forces. However, when the electroplating current becomes excessively high, the demand for nickel ions at the cathode exceeds the supply capacity from the anode electrolysis, leading to a subsequent decline in the growth trend of the weight-gain rate.

As indicated above, excessively low electroplating currents reduce the weight-gain rate and produce thinner coatings. Conversely, when the electroplating current is too high, although the weight-gain rate increases, issues such as clumping and adhesion may occur. When the electroplating current is set at 1 A and 3 A, the diamond exhibits good dispersion; however, slight adhesion appears when the current is increased to 5 A, becoming severe at 7 A. Considering the weight-gain rates observed at different electroplating current levels, the optimal electroplating current should be selected as 3 A.

### 3.2. The Influence of Output Power of an Ultrasonic Oscillator on Nickel Electroplating onto Diamond Surface

Single-factor experiments are conducted to investigate the influence of the output power of an ultrasonic oscillator on the weight-gain rate of the coating and agglomeration phenomena of diamond surfaces. The experimental conditions are as follows: diamond-loading capacity of 2 g, electroplating temperature of 50 °C, electroplating currents of 3 A, ultrasonic oscillator output power of 200 W, 500 W, and 900 W, diamond particle size of 70/80, and electroplating duration of 3 h.

**Figure 5:**
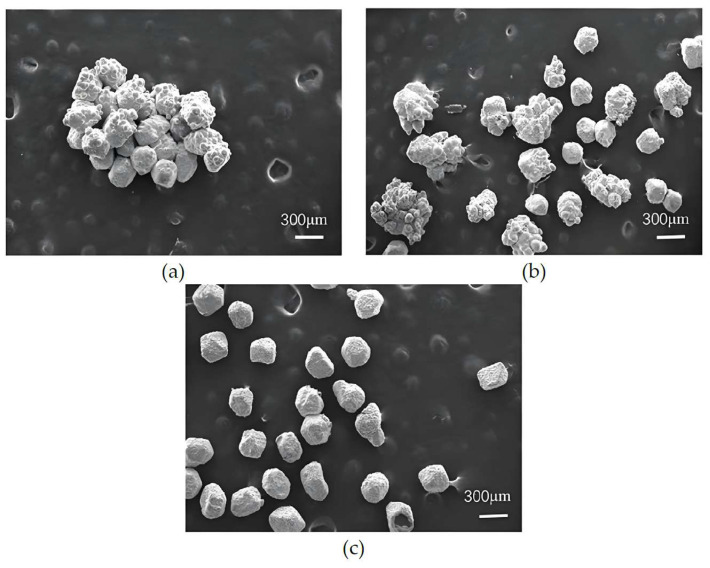
SEM images of nickel electroplating diamond under varying output power levels of ultrasonic oscillator: (**a**) 200 W; (**b**) 500 W; (**c**) 900 W.

Figure 5 presents SEM images of nickel-coated diamond particles under varying ultrasonic oscillator output power levels. Figure 5a,b demonstrate that at low output power of ultrasonic oscillator, the nickel coating on diamond surfaces becomes uneven with caking and adhesion. This occurs because, under a constant electroplating current, the deposition rate of nickel per unit time remains constant. However, when the output power of the ultrasonic oscillator is too low, some diamond particles do not move or have small motion amplitudes, leading to adhesion, agglomeration, and non-uniform plating of the diamond particles. With an increase in ultrasonic oscillator output power, the amplitude of diamond particles’ movement increases, thereby reducing the occurrence of adhesion, agglomeration, and uneven coating. However, once the output power of the ultrasonic oscillator continues to increase and reaches a critical threshold, the diamond particles around the cathode are subjected to excessive force from the ultrasonic oscillator. This can impair the contact between the diamond particles and the cathode, subsequently affecting the coating quality and the weight-gain rate of the nickel-coated diamond. This is limited by the maximum output power of ultrasonic oscillator, and the output power cannot continue to increase, so the output level of 900 W is selected as the optimal parameter. Under this experimental condition, a uniform metallic coating is observed on the surface of each diamond particle, with excellent dispersion and no signs of adhesion, as illustrated in Figure 5c.

**Figure 6:**
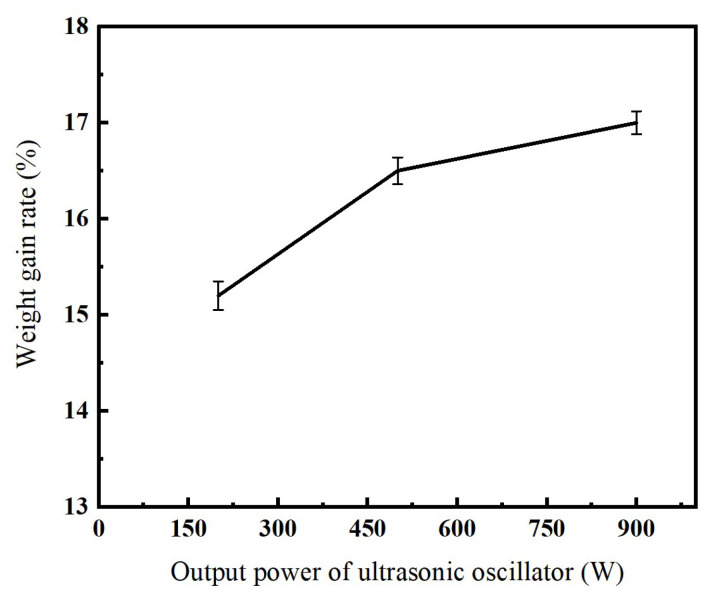
Relationship between output power of ultrasonic oscillator and coating weight-gain rate.

Figure 6 illustrates the effect of the output power of ultrasonic oscillator on the weight-gain rate of the nickel-coated diamond surface. As shown in Figure 6, the weight-gain rate increases with the output power of the ultrasonic oscillator. This phenomenon can be explained by the fact that, under a constant electroplating current, the deposition rate per unit time on the diamond particle surface is constant and insufficient ultrasonic power restricts particle mobility. Diamond particles with limited movement amplitude adhere to the cathode, reducing solution contact area and ultimately decreasing the weight-gain rate.

As the output power of the ultrasonic oscillator increases, the amplitude of the diamond particle movement also increases, which reduces the likelihood of these particles adhering to the cathode, increasing the contact area between the diamond particles and the electroplating solution, thus enhancing the weight-gain rate of the nickel-coated diamond. Experimental results confirm that ultrasonic oscillation enhances diamond particle mobility in both frequency and amplitude during electroplating. This improvement facilitates better electrical conductivity of the diamond particles and more uniform contact with the cathode, thereby contributing to improved coating quality on the diamond surface. Consequently, the method of applying a nickel electroplating coating to the diamond surface using an ultrasonic oscillator is considered technically feasible.

### 3.3. The Influence of Diamond Particle Size on Nickel Electroplating onto Diamond Surface

Single-factor experiments are conducted to investigate the influence of the diamond particle size on the weight-gain rate of the coating and agglomeration phenomena of diamond surfaces. The experimental conditions are as follows: diamond-loading capacity of 2 g, electroplating temperature of 50 °C, electroplating currents of 3 A, ultrasonic oscillator output power of 900 W, diamond particle size of 45/50, 70/80, 120/140, 230/270, and electroplating time of 3 h.

**Figure 7:**
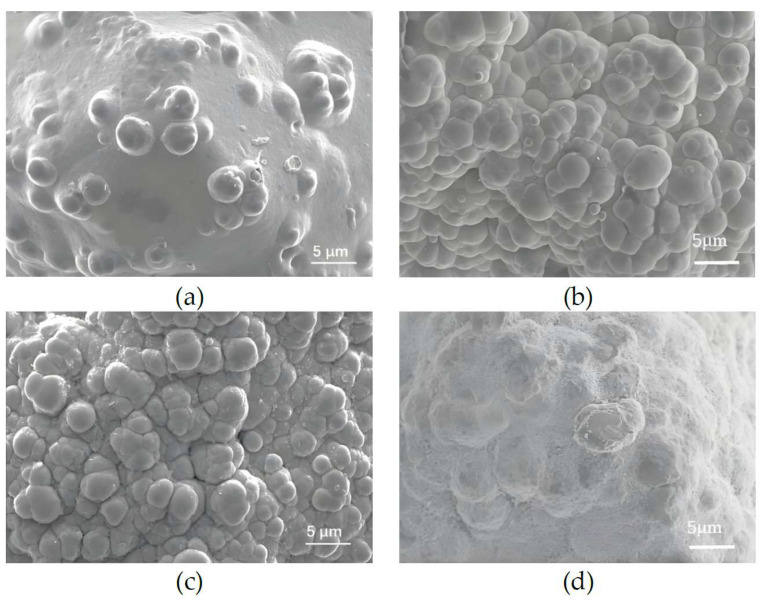
SEM images of nickel-coated diamond particles under varying diamond particle size: (**a**) 45/50; (**b**) 70/80; (**c**) 120/140; (**d**) 230/270.

Figure 7 presents SEM images of nickel-coated diamond particles under varying diamond particle sizes. It can be observed from Figure 7a–c that when the diamond particle size is 45/50, the protrusions of the coating on the diamond surface are relatively sparse. As the diamond particle size increase to 120/140, the protrusions on the surface of the coating formed on the diamond become denser, indicating an increase in the thickness of the coating. This phenomenon can be attributed to the enhanced effect of the ultrasonic oscillator on the larger diamond particles, which facilitates their movement and contact with the cathode. Consequently, this promotes the formation of new grains between larger particles, filling the gaps and resulting in a denser and smoother coating surface. However, when the diamond particle size continues to increase, as shown in Figure 7d, the protrusions of the coating on the diamond surface begin to decrease. This is primarily due to some diamond particles remaining suspended in the electroplating solution during the electroplating process, thereby failing to establish sufficient contact with the cathode. Additionally, the intensified force exerted by the ultrasonic oscillator may further reduce the effective contact between the diamond particles and the cathode, and then the coating starts to decrease.

**Figure 8:**
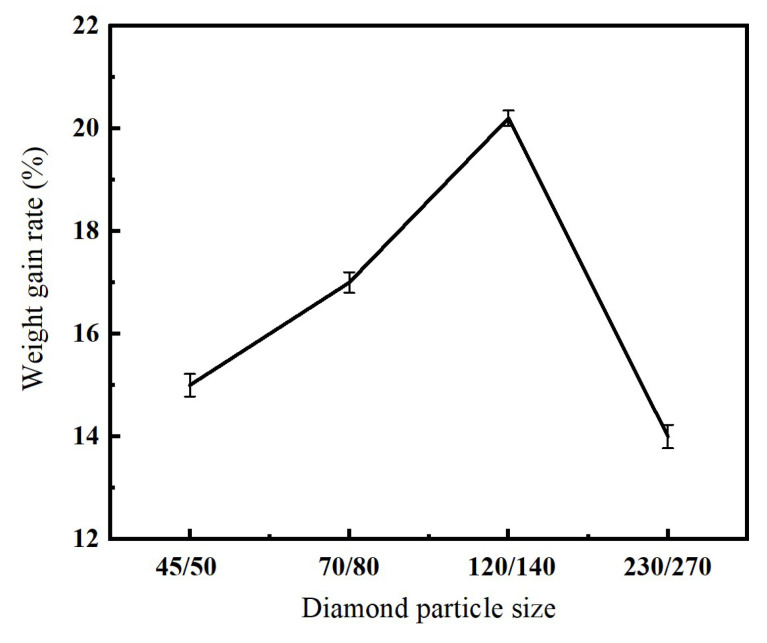
Relationship between diamond particle size and coating weight-gain rate.

As illustrated in Figure 8, diamond particle size significantly affects the weight-gain rate of the nickel-coated diamond. From the data, it can be observed that as the particle size increases, the weight-gain rate initially rises and subsequently declines. This phenomenon can be explained by two primary factors as below.

Firstly, as the diamond particle size increases, the impact of the ultrasonic oscillator during the electroplating process becomes more significant. This enhances the movement of diamond particles and their intercontact with the cathode, promoting tighter packing and thereby increasing the weight-gain rate.

Secondly, larger particle sizes result in a greater total surface area of the diamond pile, which increases the contact area with the cathode. This leads to improved current efficiency and a corresponding increase in the weight-gain rate. These two factors work synergistically to enhance the weight-gain rate. Specifically, larger diamond particles facilitate better inter-particle movement and contact, as well as increased cathode contact area, both of which contribute to a higher plating efficiency. However, when the particle size reaches 230/270, the weight-gain rate begins to decline. The main reasons for this decrease are as follows: (1) As the particle size increases further and the number of smaller particles decreases, some diamond particles may remain suspended in the electrolyte solution without making full contact with the cathode, resulting in a reduced plating rate. (2) With the increase in individual particle size and the corresponding reduction in mass, the influence of the ultrasonic oscillator becomes more intense. This can disrupt the effective contact between the diamond particles and the cathode, ultimately leading to a decline in the weight-gain rate.

Based on the aforementioned analysis, it can be concluded that during the electro-plating process, a diamond particle size of 120/140 yields the maximum weight-gain rate of 20.6%, and the coating is the densest and most uniform. Therefore, the optimal diamond particle size is recommended as 120/140.

**Figure 9:**
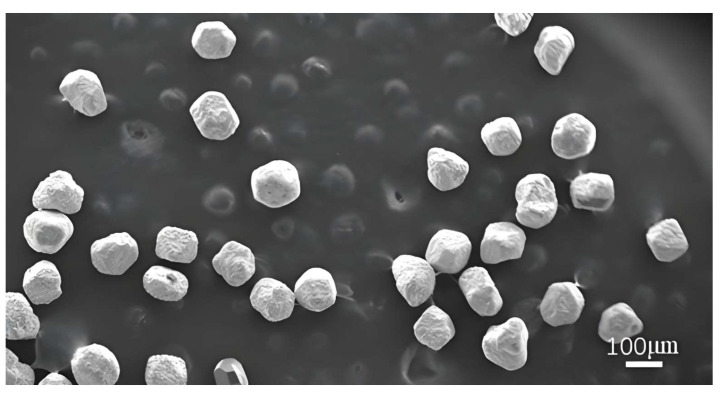
SEM image of nickel-coated diamond particles under optimal process parameters.

Considering the weight-gain rate of the coating on the diamond surface, the uniformity of the coating, as well as the presence of particle adhesion or agglomeration, the optimal process parameters are determined as follows: an electroplating current of 3 A, an ultrasonic oscillator output power of 900 W, and a diamond particle size of 120/140. Under these conditions, nickel-plated diamond with a coating rate of 20.6% is obtained. As illustrated in Figure 9, the scanning electron microscope image of the nickel-coated diamond under the optimized parameters indicates that the coating is uniformly distributed without any signs of adhesion or caking. This observation confirms the feasibility of applying the ultrasonic oscillation method for nickel electroplating on diamond surfaces.

**Figure 10:**
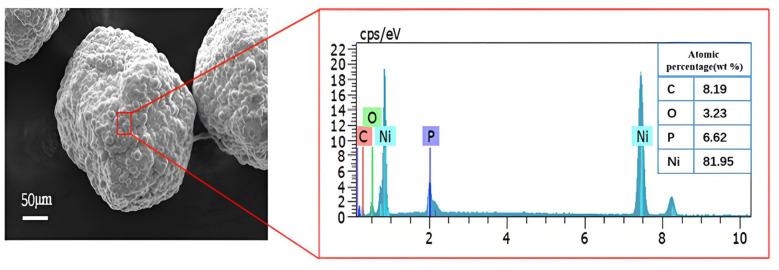
SEM image and EDS image of nickel-coated diamond particles under optimal process parameters.

Figure 10 presents the EDS energy spectrum analysis of the coating fabricated under the optimal process parameters. As shown in the figure, the Ni content of the coating reaches up to 81.95%. During the electroless nickel plating process, hypophosphite ions are reduced by hydrogen atoms present at the catalytic center’s surface and subsequently deposited on the diamond surface, resulting in a small amount of P and O in the coating. The presence of carbon atoms is attributed to carbon-containing substances, such as CO_2_ adsorb. In summary, the coating of the diamond surface prepared by this method has fewer impurities and is relatively pure.

## 4. Conclusions

In this study, an ultrasonic oscillation-assisted nickel electroplating device is developed innovatively. The effects of the output power of the ultrasonic oscillator and current density on the nickel electroplating of diamond surfaces are systematically investigated. The experimental results demonstrate the following:

(1) As the electroplating current increases from 1 A to 7 A, the weight-gain rate on the diamond surface increases correspondingly. However, when the electroplating current reaches 5 A, agglomeration and caking of diamond particles begin to occur.
(2) With the increase in the output power of the ultrasonic oscillator, the plating efficiency of nickel-coated diamonds improves significantly. When the output power is set at 900 W, the highest weight-gain rate is achieved without any signs of particle adhesion and clumping.
(3) An electroplating current of 3 A, an ultrasonic oscillator output power of 900 W, and a diamond particle size of 120/140, nickel-plated diamonds achieve a 20.6% weight-gain rate with uniform, agglomerate-free coatings.

This research demonstrates that the application of ultrasonic oscillation enhances the movement frequency and amplitude of diamond particles during the electroplating process, thereby improving inter-particle conductivity and the uniformity of contact with the cathode. Consequently, the overall quality of the nickel coating on diamond surfaces is significantly enhanced. These results further validate the effectiveness of ultrasonic oscillation in improving the uniformity of nickel coatings on diamond surfaces during electroplating.

## Figures and Tables

**Table 1:** The electroplating process variables.

Parameters	Value
Electroplating current (A)	1, 3, 5, 7
Output power of the ultrasonic oscillator (W)	200, 500, 900
Diamond particle size	45/50, 70/80, 120/140, 230/270

## Data Availability

The original contributions presented in this study are included in the article. Further inquiries can be directed to the corresponding author.
